# Surgical strategy for chest wall reconstruction secondary to cardiopulmonary resuscitation versus post-traumatic

**DOI:** 10.1007/s00068-025-02799-6

**Published:** 2025-02-28

**Authors:** Corinna Carla Dobroniak, Valeska Lesche, Ulrike Olgemöller, Paula Beck, Wolfgang Lehmann, Christopher Spering

**Affiliations:** 1 Department of Trauma Surgery, Orthopedics and Plastic Surgery, University Medical Centre Göttingen, Göttingen, Germany; 2 Department of Cardiology and Pneumology, University Medical Center Göttingen, Göttingen, Germany; 3 Department of Orthopedics and Orthopedic Trauma Surgery, Schwarzwald-Baar Hospital, Villingen-Schwenningen, Germany; 4 Department of Trauma Surgery, Orthopedics and Plastic Surgery, Universitätsmedizin Göttingen, Robert-Koch-Str. 40, D-37075 Göttingen, Germany

**Keywords:** Chest wall reconstruction (CWR), post-CPR, Chest wall instability, Sternal fracture, Serial rib fracture, Osteochondral margin

## Abstract

**Purpose:**

In mechanically cardiopulmonary resuscitated (CPR) patients, chest compressions at the level of the 3rd to 5th rib on the sternum result in reproducibly similar injury patterns: parasternal osteochondral dissociation (OCS) on both sides in combination with a sternal fracture with or without an additional serial rib fracture in the anterolateral column (ALS). This injury biomechanically impairs physiological breathing, resulting in an inverse breathing pattern. Trauma patients, on the other hand, often show a mixed pattern depending on the location of the main energy. The aim of the study was to evaluate the surgical technique of chest wall reconstruction (CWR) using transsternal refixation of the 5th rib on both sides in combination with plate osteosynthesis of the sternum and to analyze its success in comparison to the surgical strategy of CWR in the context of a traumatic genesis.

**Method:**

Data acquisition was performed using medical records of a Level I Trauma Centre in Germany and compare patients with radiologically or clinically diagnosed flail chest as a result of cardiopulmonary mechanical resuscitation (CPR). The retrospective study included patients in the period 2018–2023 after surgical CWR. The patients were either post-CPR (*n* = 29; CPR) or trauma patients (*n* = 36; trauma). The collective was described and analyzed using the digital patient file, as well as data on ICU stay and duration of ventilation or conversion to assisted ventilation modes, reason for chest wall instability, time of surgery, length of stay and mortality. As a long-term follow-up, body plethysmography was analyzed comparatively. Primary endpoints were mean length of stay in ICU, time to surgery, ventilator dependency and mortality rate. Secondary endpoints were time to transfer to rehabilitation, ventilation disorders and long term outcome.

**Results:**

In the period 65 patients (48 m, 17w) were included, 29 of whom had been mechanically resuscitated (CPR), 36 formed to post-traumatic cohort (trauma). The CPR were significantly older (69 vs. 58 years; p-value 0.003). The duration from CPR to surgery was on average significantly longer than trauma to surgery (16.76 vs. 4.11 days). The mean length of stay in ICU were 30 days (trauma) and 45 days for CPR (significantly longer, p-value 0.0008). The mean duration of ventilation was 188 h for trauma and 593 h for CPR. Extubation or conversion to assisted, relevant de-escalating ventilation modes was possible in both groups after a mean of 38 h post-OP. Among the CPR patients, 4 died in hospital (hospital mortality: CPR 20.7% vs. trauma 5.6%), 7 (30%) were transferred to an early clinical rehabilitation and 10 were discharged to home or follow-up treatment. In the case of trauma, 5 (14.7%) were transferred to an early clinical rehabilitation and 20 were discharged to home or follow-up treatment. Bodyplethysmography 6 months after CPR / trauma showed no differences in both collectives with regard to ventilation disorders. Diffusion was prolonged in both groups, presumably due to the healing process of lungs contusion. Both showed no restriction disorders.

**Conclusion:**

Chest wall reconstruction, including plate osteosynthesis of the sternum in combination with transsternal fixation of the 5th rib on both sides can largely restore physiological respiratory mechanics immediately after surgery and accelerate the weaning success. In the management of patients after CPR, the initial diagnosis which had indicated resuscitation, is the main focus and can often be an obstacle to extubation. Nevertheless, independent breathing can be accelerated by restoring the biomechanics through early surgical treatment using CWR and saves long-term ICU stays with the potential for further complication and resource consumption. CWR forms the essential basis for early rehabilitation of the underlying cause of resuscitation. Ventilation disorders do not occur after surgical CWR, even during the course of the procedure.

## Background

Around 60,000 people per year in Germany undergo CPR after an event of cardiac arrest. Current guidelines recommend chest compressions in the middle of the sternum with a frequency of 100–120/min and a depth of 5–6 cm. These compressions often lead to CPR related injuries of the chest wall, which in most cases presents as bilateral parasternal osteochondral dissociation (OCS) in combination with a sternal fracture. Often also an additional serial rib fracture in the anterolateral column (ALS) can occur.

Independently from the cause of rib fractures, their management and treatment options seem to be shifting towards surgical reconstruction if a chest wall instability occurs [8, 14, 16, 18]. While high velocity trauma remains the main cause for chest wall instability [17, 19, 22], rib fractures and sternum fractures are common injuries secondary to mechanical cardiopulmonary resuscitation (CPR) in case of cardiac arrest [1, 10, 20, 22]. The reason for cardiac arrest itself often requires complex medical care. With additional chest wall instability post- CPR medical staff faces great challenges in managing these physiologically decompensated patients. The main reason for prolonged respirator dependency after CPR seems to be a significant impairment of respiratory chest wall mechanics, leading to weaning failure [1, 17–20, 22]. Even though surgical stabilization of trauma related chest wall instability has become an important treatment strategy in the past decade, common literature only preserves a benefit if the surgical intervention is performed within the first 48–72 h after trauma [2–6, 8, 9, 12–22]. Patients post CPR often have to endure a period of physiological recompensation, before surgical chest wall reconstruction (CWR) becomes an option for further treatment [11]. A higher level of secondary complication due to chest wall instability and respirator dependency compared to trauma patients with chest wall instability can occur [7, 10, 22]. While in the past the management of these complex injuries had been mainly conservatively depending on analgesia, pulmonary toilet and pressure-controlled ventilation, chest wall instability secondary to trauma is nowadays being managed more aggressively with CWR [17, 19]. It seems to be benefitial to patients if CWR is performed soon after trauma/resuscitation. Yet standardized general protocols for indication and clinical pathways do not exist. Though regional algorithms (Fig. 4) have been implemented for trauma patients [19]. Guidelines for management of fractures that occur secondary to CPR, have not been reported in literature so far. Only a few reports analyzing this specific patient group have been published, discussing the need and benefit of CWR in post CPR injuries of the thoracic wall.

The purpose of this study was to compare patients with trauma and CPR associated injuries of the chest wall regarding different clinical parameters. Also, the study tries to emphasize the ‘typical’ injury pattern in post-CPR flail chest and establish a possible standardized surgical procedure of CWR in those injuries.

## Methods

For this study patients who underwent Chest Wall Reconstruction (CWR) between 2018 and 2023 were retrospectively analyzed. Only patients who had suffered from a chest wall injury after blunt trauma and those who underwent CWR secondary to CPR related injuries were eligible. The underlying algorithm is shown in Fig. 4 to safely perform surgery including interdisciplinary workup and measurement of hemodynamic risks as well as neurological potential outcome. All data was drawn from the digital patient records of a Level I Trauma Centre. The collected data included: Time to surgery in days, length of ICU stay (ICU-LOS), length of stay in the Level I Trauma Centre, total time of respirator dependency, time of de-escalation of ventilation post CWR (transition to assisted ventilation mode) / extubation and 30-day mortality rate. The exact injury pattern of each patient was detected from chest CT scans after trauma/CPR and the documentation from the actual surgery. All surgeries were performed using the MatrixRIB Fixation System (Depuy Synthes, Oberdorf, Switzerland).

For traumatic injuries depending on the location of flail segments plate osteosynthesis and intramedullary splints were used. In post CPR patients the operative strategy according to our protocol is always similar: plate osteosynthesis of the sternum and a bilateral transsternal fixation of either the 4th or 5th rib. If there were more fractures detected in the anterolateral column of the thoracic wall plate- or splint osteosynthesis was added (Fig. 2 and 3).

Furthermore, according to our institution’s protocol patients who underwent CWR after injury of the thoracic wall were assigned to bodyplethismography 6 months after surgery. If available the results were added to the data collection as well.

## Results

### Patient characteristics

A total number of 65 patients was included in the study. 48 of the included patients were male, while17 female patients were recorded. 36 patients were reported to the group of CWR after trauma and 29 to the group of post-CPR CWR. Descriptive analyses showed that patients from the post CPR group were significantly older (Table 1; 69 vs. 58 years; p-value 0.003).

### Pattern of injury

Depending on a variety of circumstances, injury of the thoracic wall after high velocity trauma can appear in all columns of the thoracic wall including the sternum. Typical fracture pattern of the chest wall has been recently investigated and they are summarized in recent publications [17, 19].

**Fig. 1:**
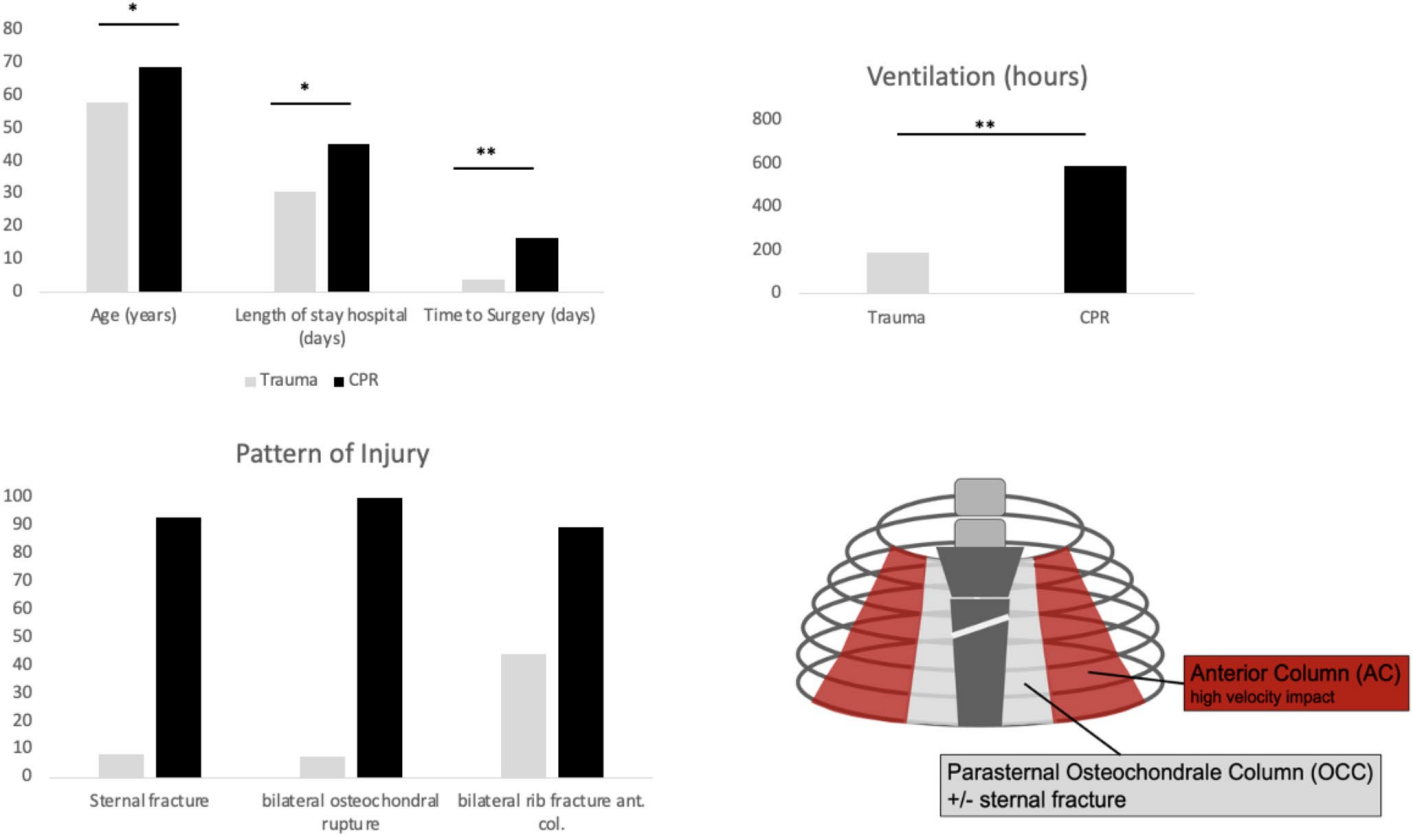
Comparison of the two groups: Trauma versus CPR. Shown are significant differences between the groups in regard of age, hospital LOS and time to surgery as well as total ventilation hours. The injury pattern shows clear difference in regard of the proportion of sternal fractures and bilateral osteochondral dissociation. The affected columns of the chest wall are shown in the drawing to visualize the typical pattern

**Fig. 2:**
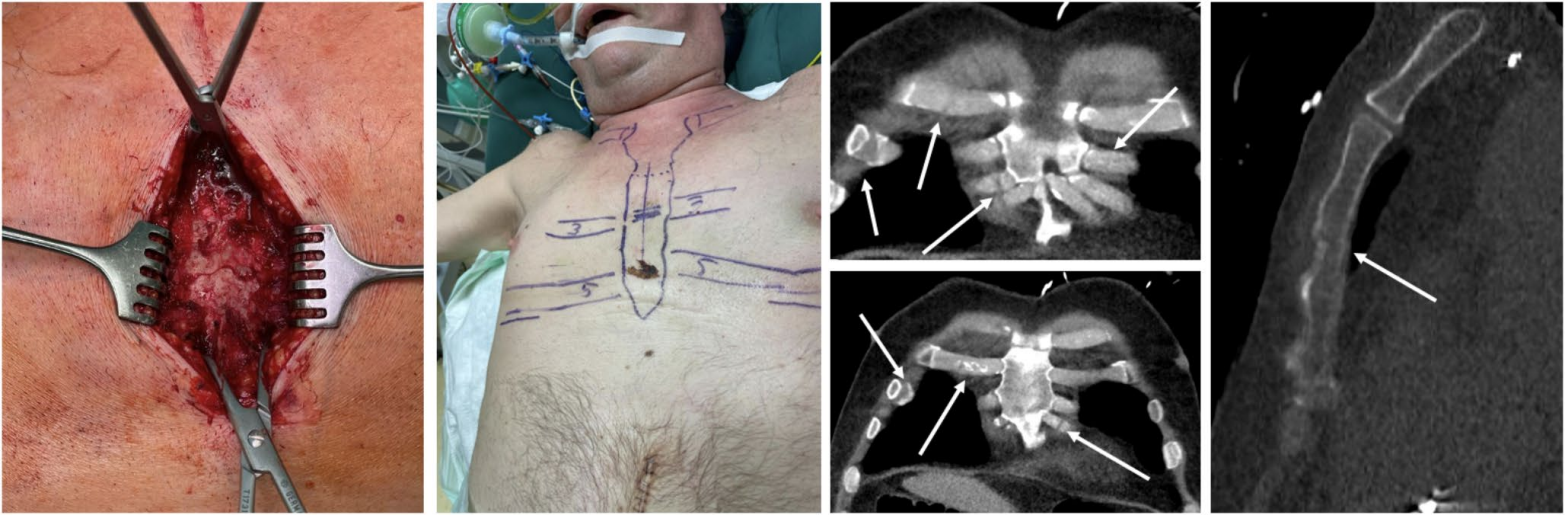
The standard fracture pattern is an undisplaced sternal fracture (left picture) and the osteochondral parasternal dissociation both sides, which can be difficult to detect in CT-diagnostics. The fracture pattern is then “mapped” on to the patient, to plan the typical three incisions, as shown in the picture in the middle

**Fig. 3:**
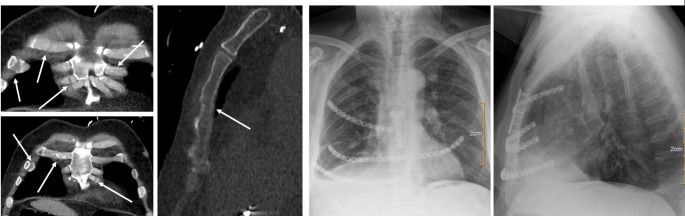
The typical CT-scan findings are usually difficult to find, since the sternal fracture is undisplaced and the osteochondral dissociation needs special attention. The surgical strategy addresses the injury pattern: plating osteosynthesis of the sternum + transsternal fixation of at least the 5th rib on both sides. If additional fractures are displaced in the anterior or antiolateral column, additional plating can be beneficial as seen in the postOP x-ray

Apart from that, injuries to the thoracic wall post CPR follow a characteristic pattern in most cases. All 29 patients included in the post CPR group showed bilateral parasternal osteochondral dissociation, 27 (93%) in combination with a sternal fracture. In addition, most patients post CPR presented with additional serial rib fractures in the anterolateral column of one or both sides (Fig. 1). The typical pattern of injury is not easy to detect in CT diagnostics, due to usually undisplaced fractures and osteochondral dissociation. Figures 2 and 3 show the typical clinical finding and surgical strategy.

### Management of patients and surgery

Preoperatively all patients were admitted to an ICU. The mean length of stay on ICU were 30 days for the trauma group and 45 days for post-CPR patients (p-value 0.008). The time span in between the event of injury (Trauma/CPR) and surgery was significantly longer in the post-CPR group (16.76 vs. 4.11 days; Table 1). Similar to that the mean duration of ventilation in post CPR patients (593 h) was significantly longer than in patients of the trauma group (188 h) (Fig. 1). The duration between surgery and extubation/ relevant de-escalation of ventilation mode post-operatively showed no significant difference with 38 h on average in both groups.

**Table 1:** Comparison of patients after Chest Wall Reconstruction secondary to CPR versus trauma

	CPR	Trauma	*p*-value
*N* = 65 f: 17 m: 48	29	36	
Age [years]	69	58	0.003
Time to surgery [days]	**16.76**	**4.11**	< 0.001
ICU-LOS [days]	**45**	**30**	< 0.001
Ventilation [h]	**593**	**188**	**0.004**
postOP Extub. [h]	**37.9**	**37.5**	**n.s.**
in-hospital mortality rate [%]	**20.7**	**5.6**	
Discharge to early interv. rehab	**7 (30%)**	**5 (14.7%)**	

### Outcome and discharge

In the post-CPR group 4 patients (20.6%) died after surgery on ICU, while in the trauma group a total of 2 patients (5.6%) passed away. Among the patients of the post-CPR group 7 (30%) were transferred to an early clinical rehabilitation unit and 10 patients were discharged home or to follow-up rehabilitation treatment. In patients of the trauma group, 5 (14.7%) were transferred to an early clinical rehabilitation unit while 20 were discharged home or follow-up rehabilitation treatment. Not all patients were eligible to perform bodyplethysmography 6 months after CWR. In those available results no difference in both groups in regard to ventilatory function could be detected. Diffusion was prolonged in both groups, presumably due to the healing process of lungs contusion. Both showed no restriction disorders.

## Discussion

The correct and careful indication as well as the timing of surgical reconstruction are decisive for the patient’s outcome. Current literature shows that patients benefit if they suffer from a radiologically and/or clinically unstable chest wall and are operated on within the first 24–48 h. However, a change of procedure can also make sense for patients who have initially been treated conservatively if the previous measures have failed. However, in order to achieve a significant positive effect on mortality and morbidity, the decision should be made within the first 48 h [2–6, 13, 15–19, 21]. If patients cannot be weaned off the respirator after prolonged conservative therapy and if biomechanical reasons can be attributed to this circumstance, surgical treatment should still be considered at a later stage. Patients who suffer from chest wall instability secondary to CPR belong to the delayed treatment group due to the underlying medical condition which quite often results in postponed surgical procedures.

The above mentioned data and other results, mostly from retrospective studies, further support the trend [5, 11–16, 18, 21] and led to the publication of consensus statements for thoracic osteosynthesis [13–16]. But there has not been broad research on CPR patients and most available data only result from case reports [1]. In a recently published meta analysis [22] the authors come to the conclusion, that surgical rib fixation does not seem to be very common, although chest wall instability and prolonged respiratory dependencies with the entire spectrum of complication are seen.

However, it should not be forgotten that conservative treatment concepts with complex pain management such as peridural anaesthesiological procedures and selective intercostal nerve blocks, bronchial lavage, tracheotomy and mechanical pressure-controlled ventilation with increased Positive EndExpiratory Pressure (PEEP) have not lost their importance. The vast majority of chest wall injuries are still treated conservatively despite the cause of instability (trauma or post-CPR) and despite the known risk of prolonged stay on intensive care, long-term ventilation and pulmonary complication. In case of an unstable chest wall pneumonia rate, ventilation time and length of stay on intensive care can be positively influenced with early surgical stabilization [13–17, 19, 20]. An additional sternal fracture is considered a significant factor for inverse breathing and biomechanical chest wall instability and should therefore also be stabilized on a case-by-case basis.

**Fig. 4:**
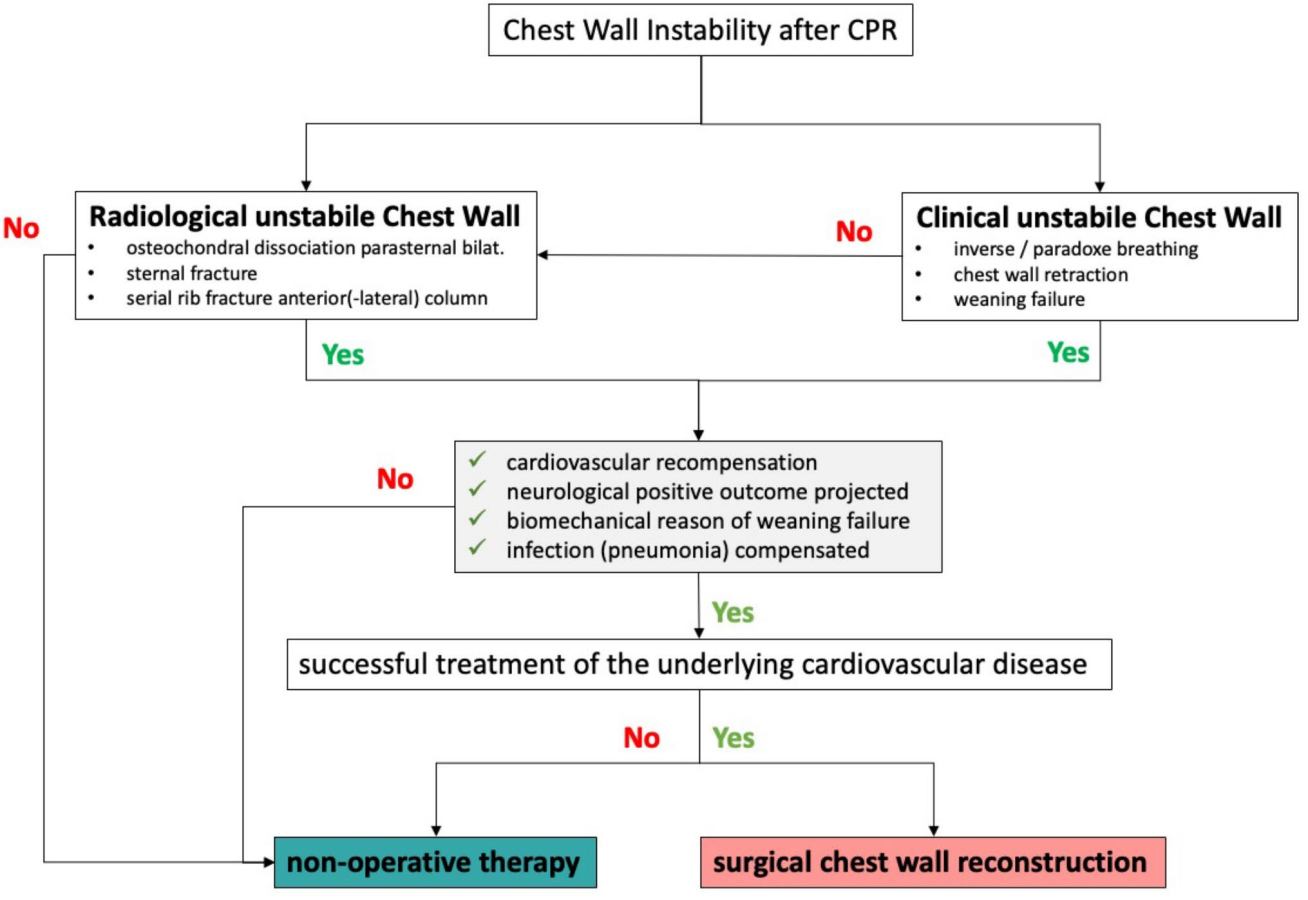
Algorithm for safe surgical reconstruction of a chest wall instability secondary to cardiopulmonary resuscitation

Detailed preparation and planning of the procedure prior to surgery is of the essence especially in patients post-CPR. Open reduction and minimally invasive osteosynthesis using intraoperatively individualized precontured, low profile locking plates and / or intramedullary splints provide minimally invasive surgical strategies. But the patient still needs to be hemodynamically stable enough to undergo surgery. Therefore the underlying algorithm (Fig. 4) is especifically designed for these kind of patients and addresses the status of the patient in respect of the underlying medical condition: (1) cardiovascular recompensation, (2) Neurological positive outcome projected, (3) Biomechanical reason of weaning failure and (4) Infection (pneumonia) compensated (Fig. 4). Thus, the algorithm in post-CPR patients differs significantly from published post-traumatic algorithms for CWR [15, 19]. Continuous communication with the anaesthesiologist during surgery regarding hemodynamical and respiratory status of the patient is crucial. The neurological potential should be tested prior to surgery in case of post-CPR patients through a wake-up, to avoid surgery in patients with poor or no positive neurological outcome.

Immediate weaning after surgery should be possible regarding the stability of the chest wall. The provided data shows the rapid extubation or de-escalation after chest wall reconstruction. Although the fractures might have healed without any surgery, the important rehabilitation of the underlying medical condition, which had initially indicated CPR, can be performed earlier when the unstable chest wall has been reconstructed and the patient has successfully been weaned from the respirator.

Although a transsternal plating strategy might not seem to be the best choice of stabilization, usually implant removal is not necessary.

## Conclusion

Chest wall reconstruction, including platePlate osteosynthesis of the sternum in combination with transsternal fixation of the 5th rib on both sides can largely restore physiological respiratory mechanics immediately after surgery and accelerate the weaning success. In the management of patients after CPR, the initial main diagnosis which had indicated leading to resuscitation, is the main focus and can often be is an obstacle to extubation. Nevertheless, independent breathing can be accelerated by restoring the biomechanics through early surgical treatment using CWR and saves longer-term ICU stays with the potential for further complications and resource consumption. CWR forms the essential basis for early rehabilitation of the underlying cause of resuscitation. Ventilation disorders do not occur after surgical CWR, even during the course of the procedure.

## Limitations

The provided data set is limited to a single centre, retrospective study. The patient selection has not been blinded but performed via medical algorithm (Fig. 4) and medical condition. Nevertheless the medical workup prior to surgery has been performed interdisciplinary to safely operate on these patients. Thus the comparison between trauma patients and patients post-CPR can only be performed relatively in regard of limited outcome parameters. Time of respirator dependency and length of stay at ICU is not only depending on the chest wall instability. Therefore the provided data can only deal as recommendations, further prospective randomized multicentre studies are needed.

## Data Availability

The underlying data is provided within the manuscript.
